# Assessing Interactions of Two Loci (rs4242382 and rs10486567) in Familial Prostate Cancer: Statistical Evaluation of Epistasis

**DOI:** 10.1371/journal.pone.0089508

**Published:** 2014-02-25

**Authors:** Li-Sheng Chen, Jean Ching-Yuan Fann, Sherry Yueh-Hsia Chiu, Amy Ming-Fang Yen, Tiina Wahlfors, Teuvo L. Tammela, Hsiu-Hsi Chen, Anssi Auvinen, Johanna Schleutker

**Affiliations:** 1 School of Oral Hygiene, College of Oral Medicine, Taipei Medical University, Taipei, Taiwan; 2 Department of Health Industry Management, School of Healthcare Management, Kainan University, Tao-Yuan, Taiwan; 3 Department and Graduate Institute of Health Care Management, College of Management, Chang Gung University, Tao-Yuan, Taiwan; 4 Institute of Biomedical Technology/BioMediTech, University of Tampere, Tampere, Finland; 5 Department of Urology, Tampere University Hospital, and School of Medicine, University of Tampere, Tampere, Finland; 6 Graduate Institute of Epidemiology and Preventive Medicine, College of Public Health, National Taiwan University, Taipei, Taiwan; 7 Tampere School of Public Health, University of Tampere, Tampere, Finland; 8 Department of Medical Biochemistry and Genetics, Institute of Biomedicine, University of Turku, Turku, Finland; University of Nebraska Medical Center, United States of America

## Abstract

Understanding the impact of multiple genetic variants and their interactions on the disease penetrance of familial multiple prostate cancer is very relevant to the overall understanding of carcinogenesis. We assessed the joint effect of two loci on rs4242382 at 8q24 and rs10486567 at 7p15.2 to this end. We analyzed the data from a Finnish family-based genetic study, which was composed of 947 men including 228 cases in 75 families, to evaluate the respective effects of the two loci on the disease penetrance; in particular, the occurrence and number of prostate cancer cases within a family were utilized to evaluate the interactions between the two loci under the additive and multiplicative Poisson regression models. The risk alleles A at rs4242382 (OR = 1.14, 95% CI 1.08–1.19, P<0.0001) and a risk allele A at rs10486567 (OR = 1.06, 96%CI 1.01–1.11, P = 0.0208) were found to be associated with an increased risk of familial PrCa, especially with four or more cases within a family. A multiplicative model fitted the joint effect better than an additive model (likelihood ratio test X^2^ = 13.89, P<0.0001). The influence of the risk allele A at rs10486567 was higher in the presence of the risk allele A at rs4242382 (OR = 1.09 (1.01–1.18) vs. 1.01 (0.95–1.07)). Similar findings were observed in non-aggressive PrCa, but not in aggressive PrCa. We demonstrated that two loci (rs4242382 and rs10486567) are highly associated with familial multiple PrCa, and the gene-gene interaction or statistical epistasis was consistent with the Fisher's multiplicative model. These loci's association and epistasis were observed for non-aggressive but not for aggressive tumors. The proposed statistical model can be further developed to accommodate multi-loci interactions to provide further insights into epistasis.

## Introduction

Genetic predisposition and familial aggregation of prostate cancer (PrCa) have been demonstrated in numerous studies; a twin study showed a very high heritability score [1]. Men with one affected first-degree relative have a two-fold increased risk of PrCa and even higher risk for an early onset of PrCa compared to those without such a relative [2], [3]. The recent genome-wide association studies have identified multiple genetic variants in over 40 loci that are significantly associated with a risk of prostate cancer [4]. Originally, these variants were mainly found in altogether five chromosomal regions; three independent regions of 8q24, in one region of 17q12, and one region of 17q24.3[5]–[9]. However, it has been reported that a family history is predictive for the risk of prostate cancer independently of the effect of SNPs in the risk associated chromosomal regions [1]. In addition to the regions on chromosomes 8 and 17, a specific SNP in the *JAZF1* gene at 7p15.2 has been repeatedly associated with PrCa risk [10]–[13]. Besides overall risk, of particular interested are its reported associations with early onset, and aggressive disease, as well as with biochemical recurrence, suggesting prognostic importance [14]–[16].

The SNP known as rs10486567 is located within the intron 2 of the *JAZF1*, which encodes a transcriptional repressor of NR2C2, a nuclear orphan receptor that is highly expressed also in prostate cancer [13].

Because a number of SNPs are involved in familial risk for prostate cancer, but their independent main effects explain only a fraction of the observed heritability, gene-gene interaction between loci (departure from independence of effects, which is known as epistasis in genetics and effect modification in epidemiology) provides a potential improvement in understanding the hereditary component of prostate cancer [17]. Of the identified SNPs, we selected two SNPs, a common and consistent risk SNP rs4242382 within 8q24 on chromosome 8, and the SNP rs10486567 within *JAFZ1* on chromosome 7 with consistent associations in various populations and also with different disease outcomes.

We aimed to evaluate the impact of two loci on rs4242382 and rs10486567 on the prostate cancer risk within a family. We also evaluated interactions between the two loci in additive and multiplicative models. Separate analyses were also conducted for aggressive and non-aggressive PrCa.

## Materials and Methods

### Data Sources and study design

**Figure 1:**
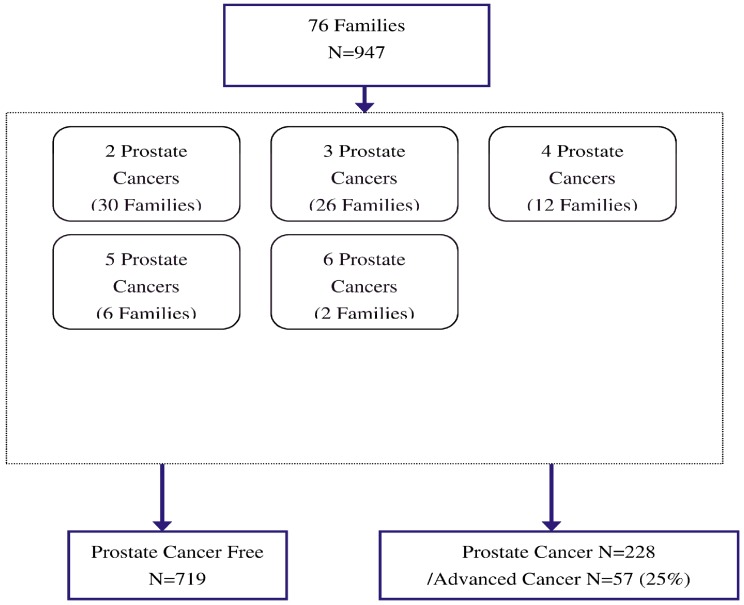
Family members with PrCa among 76 Finnish families.

The data used for the following analysis are from a population-based cohort that consisted of patients diagnosed with prostate cancer in the Pirkanmaa Hospital District and control subjects selected from the anonymous male blood donors obtained from the Finnish Red Cross. The study design and the DNA sample collection have been described in previous studies [18], [19]. The data used for the familial aggregation analysis of PrCa were derived from a Finnish family study that enrolled 947 subjects from 76 families with 2–6 family members (Figure 1). We used a family-based study design by dividing these 947 subjects into 719 unaffected relatives and 228 cases with PrCa. The oldest unaffected cases were selected for controls from each family. The mean ages were 61.5 and 65.0 for unaffected relatives and cases, respectively. Among the 228 patients with prostate cancer, 25% (N = 57) were clinically advanced and had Gleason Score≥7; they were classified as aggressive cancers. Through these index cases, the total of 228 prostate cancer cases among family members were found to have an outcome following a Poisson regression model with the genotypes for the two loci, rs4242382 at 8q24 and rs 10486567 at 7p, defined as the independent variables.

### Genotype

Two loci, rs4242382 at 8q24 and rs 10486567 at 7p15.2, with genotypes AA, GA, and GG, were selected for the analysis for the reasons outlined above. The risk allele A of rs4242382 at 8q24 has been previously reported to be associated with an aggressive PrCa [2], [8], [19]–[24]. The risk allele G of rs 10486567 at 7p15.2 on the intron 2 of the JAZF zinc finger1 gene (*JAZF1*) is commonly observed in the Europeans [9].

### Statistical Analysis

The frequencies of the two SNPs were expressed as percentages. The frequencies of the genotype AA or GA versus GG are listed by the number of affected men for the two loci. By taking the number of PrCa cases among the family members of a proband as the outcome, we used a multi-variable Poisson regression model to evaluate the effect of the genotypes AA/GA versus GG for the two SNPs on the number of PrCa cases in the family. In addition, we evaluated the gene interactions between the two SNPs under the two models of statistical epistasis, the additive model and multiplicative model proposed by Fisher. We used the likelihood ratio test with Akaike Information Criterion (AIC) measures to assess whether an additive or a multiplicative model fitted the data better when it included the two loci vs. only one locus.

## Results

Figure 1 shows the families of 947 study subjects, where 719 of them were healthy and 228 diagnosed with PrCa. They included 30 families with two members with PrCa, 26 families with three, 12 families with four, six families with five, and two families with six family members diagnosed with PrCa.

The allele frequencies were calculated as 6.4% of AA (n = 61), 31.8% of GA (n = 301), and 61.8% of GG (n = 585) for rs4242382; 6.5% of AA (n = 61), 36.9% of GA (n = 348), and 57.6% of GG (n = 534) for rs10486567.

The frequency of the risk allele A (AA or GA) at rs4242382 increased from 34.2% for families with two PrCa cases up to 53% for family families with at least five affected members (Table 1). Similarly, the frequency of the risk allele A at rs10486567 increased from 42.2% for families with two affected members to 49.4% for those with five or more cases. An equally strong relation was not found for the risk allele frequencies and the number of aggressive PrCa cases.

**Table 1:** Number of total prostate cancer cases and aggressive prostate cancer cases among the family members stratified by the alleles for rs4242382 & rs10486567 loci.

	SNP-rs4242382				SNP-rs10486567			
No. of PrCa cases among family members	GG		AA or GA		GG		AA or GA	
All PrCa	N	%	N	%	N	%	N	%
2	198	65.8	103	34.2	174	57.8	127	42.2
3	228	71.7	90	28.3	193	61.1	123	38.9
4	79	49.7	80	50.3	82	51.9	76	48.1
5+	80	47.3	89	52.7	85	50.6	83	49.4
	P<0.0001				P = 0.0383			
Aggressive PrCa								
0	265	60.5	173	39.5	257	58.9	179	41.1
1	209	69.0	94	31.0	163	54.0	139	46.0
2	97	53.6	84	46.4	99	55.0	81	45.0
3	14	56.0	11	44.0	15	60.0	10	40.0
	P = 0.006				P = 0.546			
Total	585		362		534		409	

In the Poisson regression analysis considering age as a confounding factor, the risk allele A at rs4242382 was associated with an increased risk of familial multiple PrCa cases (aOR = 1.19, 95% CI 1.08–1.19, P<0.0001, Table 2). In addition, the risk allele A at rs10486567 showed a significant but slightly weaker effect (aOR = 1.06, 95% CI 1.01–1.11, P = 0.0208).

**Table 2:** The additive model of an association between the two SNPs and the risk of prostate cancer.

		One Loci			Two Loci		
					Additive Model		
Cancer Type	SNPs	Coefficient	OR (95%CI)	*p-Value*	Coefficient	aOR (95%CI)	*p-Value*
Prostate Cancer							
	Intercept	1.1878			1.1707		
	rs4242382 (AA or GA vs GG)	0.1271	1.1355	<0.0001	0.1245	1.1326	<0.0001
			(1.0840–1.1895)			(1.0808–1.1867)	
	Intercept	1.2053					
	rs10486567 (AA or GA vs GG)	0.0550	1.0560	0.0208	0.0424	1.0433	0.0727
			(1.0084–1.1071)			(0.9961–1.0927)	
Non-aggressive Prostate Cancer							
	Intercept	1.0608			1.0469		
	rs4242382 (AA or GA vs GG)	0.1462	1.1575	<0.0001	0.1453	1.1564	<0.0001
			(1.0776–1.2433)			(1.0759–1.2429)	
	Intercept	1.0800					
	rs10486567 (AA or GA vs GG)	0.0484	1.0496	0.1858	0.0336	1.0342	0.3562
			(0.9770–1.2758)			(0.9629–1.1109)	
Aggressive Prostate Cancer							
	Intercept	−0.5249			−0.5462		
	rs4242382 (AA or GA vs GG)	0.0646	1.0667	0.4101	0.0579	1.0596	0.4634
			(0.9147–1.2439)			(0.9076–1.2371)	
	Intercept	1.0800					
	rs10486567 (AA or GA vs GG)	0.0591	1.0609	0.4446	0.0533	1.0548	04927
			(0.9117–1.2345)			(0.9057–1.2284)	

When the two loci were considered simultaneously in an additive model, the regression coefficients were slightly decreased from aOR = 1.14 (1.08–1.19) to aOR = 1.13 (1.08–1.19) for the risk allele A at rs4242382, as well as from aOR = 1.06 (1.01–1.11) to aOR = 1.04(1.00–1.09) for the risk allele A at rs10486567. Adding either risk allele improved the fit of the model at a statistically significant level compared with a single locus model (risk allele A at rs4242382 resulted in X^2^_(1)_ = 183.0093, P<0.0001, and the A at rs10486567 with X^2^_(1)_ = 16.89, P<0.0001, Table 1), which suggests that the effects of the two risk alleles were independent in the context of the additive model. Comparable results were observed for non-aggressive PrCa, albeit the risk allele A at rs4242382 was more influential than the risk allele A at rs10486567 with the latter being non-significant (P = 0.74). For the aggressive PrCa, no significant improvement was found to the single locus model when the two-loci model were used (P-values 0.46 and 0.49).

The multiplicative model with an interaction term for the two SNPs fitted the data significantly better than the corresponding additive or multiplicative models (P = 0.0002, Table 3; AIC = 7713.23, see Table S1 in File S1). A significant improvement was also observed for the data from non-aggressive PrCa, but not from the aggressive PrCa. The effect of the risk allele A at rs4242382 was modified by the risk allele A at rs10486567, as shown in Table 3 along with the results of each locus stratification by the other risk allele A for PrCa and non-aggressive PrCa. The effect of the risk allele A at rs10486567 was stronger in the presence of the risk allele A at rs4242382 (aOR = 1.09, 1.01–1.18 vs. 1.01, 0.95–1.07), which indicates a synergistic epistasis (Table 4). A similar finding was observed for the effect of rs4242382 in relation to rs10486567 (aOR = 1.18, 1.10–1.27, in the absence of the latter vs. 1.09, 1.02–1.16, when carrying both). Such an effect on risk modification between the two loci was also observed for non-aggressive PrCa.

**Table 3:** The multiplicative model vs. the additive model of an association between the two SNPs and the risk of prostate cancer.

	Prostate Cancer		Non-aggressive Prostate Cancer		Aggressive Prostate Cancer	
SNPs	Coefficient	p-Value	Coefficient	p-Value	Coefficient	p-Value
Intercept	1.1815		1.0586		−0.5446	
rs4242382 (AA or GA vs GG)	0.0872		0.0992		0.0521	
Intercept						
rs10486567 (AA or GA vs GG)	0.0085		−0.0088		0.0483	
Interaction	0.0799	0.0002	0.0988	0.0497	0.0125	0.5445

**Table 4:** Stratified odds ratios for rs10486567 and rs4242382 with respect to the risk of prostate cancer.

Stratum	SNPs	Odds ratio
**Prostate Cancer**		
Stratified by rs4242382		
AA or GA	rs10486567 (AA or GA vs GG)	1.09(1.01–1.18)
GG	rs10486567 (AA or GA vs GG)	1.01(0.95–1.07)
Stratified by rs10486567		
AA or GA	rs4242382(AA or GA vs GG)	1.18(1.10–1.27)
GG	rs4242382(AA or GA vs GG)	1.09(1.02–1.16)
**Non-aggressive Prostate Cancer**		
Stratified by rs4242382		
AA or GA	rs10486567 (AA or GA vs GG)	1.09(0.98–1.23)
GG	rs10486567 (AA or GA vs GG)	0.99(0.90–1.08)
Stratified by rs10486567		
AA or GA	rs4242382(AA or GA vs GG)	1.22(1.09–1.37)
GG	rs4242382(AA or GA vs GG)	1.10(1.01–1.21)

**Figure 2:**
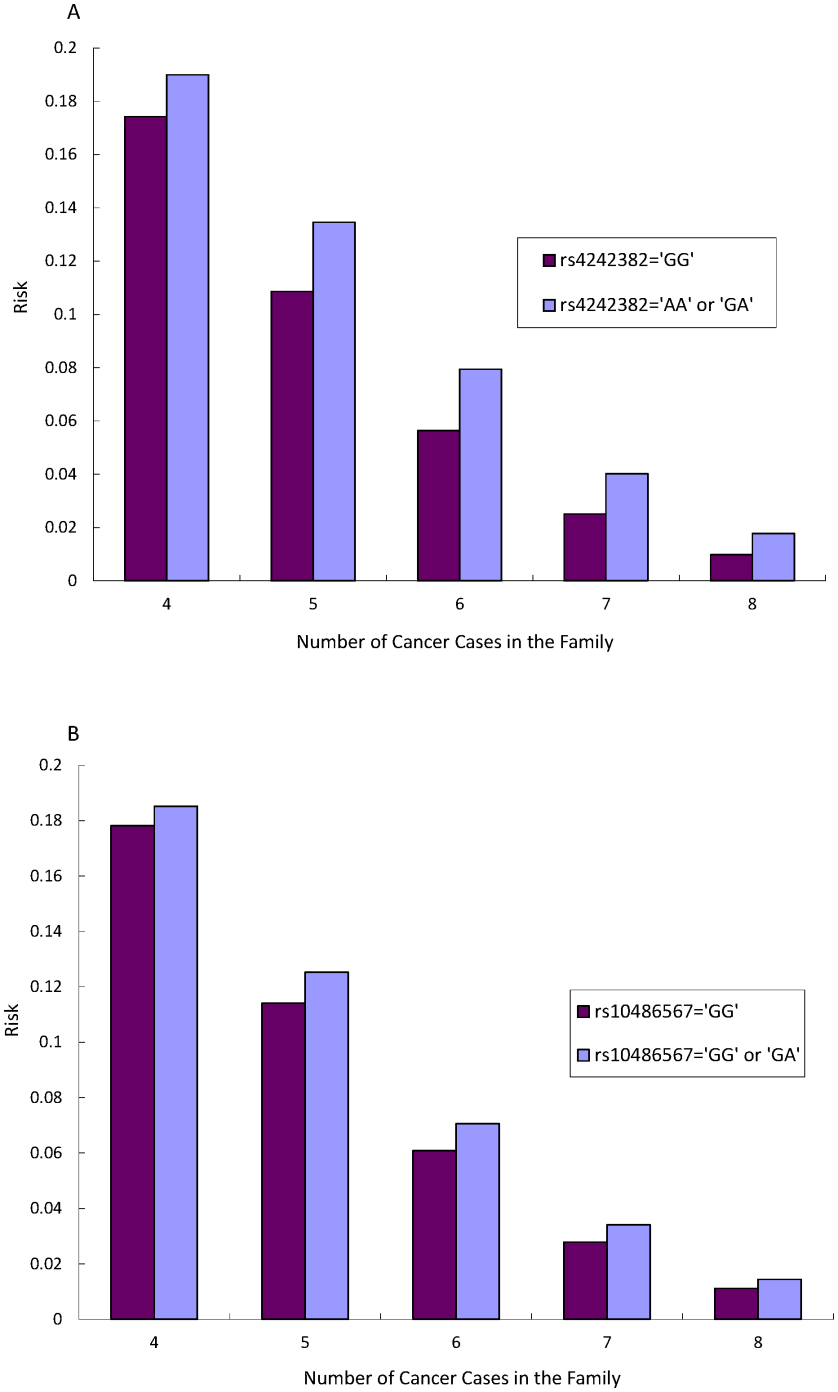
A. The risk of having multiplex prostate cancer families with the SNP rs4242382. B. The risk of having multiplex of prostate cancer cases in a family for the SNP of rs10486567

**Figure 3:**
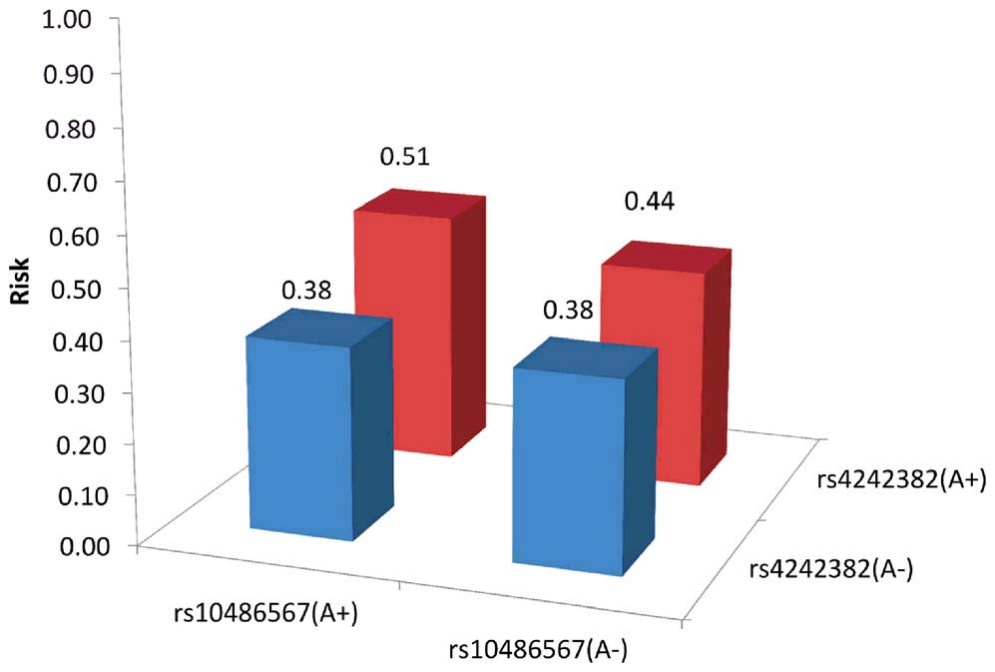
Risk of having four or more prostate cancer cases among family members with the SNPs rs4242382 and rs10486567.

Figure 2 shows the probability of having at least four PrCa cases among family members predicted by the Poisson regression model. Those who had the risk allele A at both rs4242382 and rs10486567 showed higher probability of having at least four affected relatives. Figure 3 also shows the probabilities of having at least four PrCa cases among family members in combination with having the risk allele A at rs4242382 and at rs10486567. Those carrying the risk allele for both loci had a 13% higher risk for having at least four PrCa cases among family members than those not carrying the risk allele.

## Discussion

In spite of numerous studies addressing genetic susceptibility to prostate cancer, very few studies have been conducted to evaluate the effects interactions (epistasis) on the disease penetrance by using the state-of-the-art statistical analysis of joint effects. Furthermore, our end-point was multiple PrCa cases within a family, which has rarely been studied. Epistasis in genotype level is defined as the interaction among multiple genes or loci, and this joint genetic effect may be the factor behind “missing heritability”, a phenomenon linked to the unexplained portion of hereditary cancer susceptibility, which is observed in PrCa. In the current study, we used family-based data to investigate the effect of two loci, rs4242382 at 8q24 and rs10486567 at 7p15.2, on multiple PrCa cases within families with the Poisson regression method to compare additive and multiplicative models. We demonstrated a statistically significant association between the two individual loci and multiple PrCa, as well as a synergistic gene-gene interaction between the two risk alleles. Gene-gene interactions were statistically significant under both models, but the multiplicative model provided a better fit than the additive model with respect to the likelihood ratio test with the AIC criterion. The genetic interactions (joint effect of rs4242382 at 8q24 and rs10486567 at 7p15.2) resulted in a positive statistical epistasis (enhancement) in the multiplicative model but a slightly negative epistasis (antagonistic effect) in the additive model. This statistical epistasis was also observed for non-aggressive PrCa, but not for the aggressive PrCa.

Our findings for rs4242382 at 8q24 and rs 10486567 at 7p15.2 were consistent with the genome-wide study in which the risk allele A of rs4242382 at 8q24 led to a 41% increase in the risk of non-aggressive PrCa and a 66% risk increase in the aggressive PrCa compared with the control group; the risk allele G of rs10486567 at 7p15.2 was associated with a 18% decrease in non-aggressive PrCa and 8% decrease in the aggressive PrCa [13]. The association between rs4242382 at 8q24 and the risk for prostate cancer has been consistently reported in a number of genome-wide studies [2], [8], [20]–[24]. The risk allele G of rs10486567 is reported as the major allele in the Europeans. In our study, the frequency of the risk allele G was approximately 75%. However, the direction of the association between rs10486567 at 7p15.2 and the risk of prostate cancer has not been consistent in multiple studies. The results published by Thomas et al. [13] as well as our study, indicate an inverse association; in contrast, several others have shown a positive association [15], [24]. The SNP rs10486567 located within intron 2 of *JAZF*1 gene on chromosome 7p15.2 encodes a three C2-H2-type zinc finger protein, which is a transcriptional repressor of NR2C2, a nuclear orphan receptor that is highly expressed in prostate tissue and interacts with the androgen receptor. There is no biological interpretation for the functional implications of JAZ1 in prostate carcinogenesis. It has been reported that *JAZF*1 is a component of gene fusion with *SUZ*12, which is found in endometrial stromal tumor. The inverse association found in both Thomas's and our study may be due to the increased risk for T2D, which has been reported to be inversely associated with PrCa [13]. The area on 8q24 is associated to many cancers, for example breast, colon and bladder cancer, in addition to PrCa, and the area is shown to have multiple regulatory variants. Therefore, it is possible that the SNPs analyzed here or other SNPs in linkage disequilibrium near the tested ones affect the expression of the gene they resided in, possibly acting as regulators for the other gene. Also, *JAZF1* is known to have alternatively spliced variants, which encode for different protein isoforms but not all variants have been fully characterized. These may be tissue type and/or SNP specific. However, no explicit conclusions about the interactions between these variants can be made without further functional validation. The results presented here were from a relatively small sample set and therefore additional studies are warranted, also in other populations.

A family-based study design is ideal for assessing the independent genetic influence of several SNPs and their joint effects with other genetic determinants on the disease penetrance among multiple PrCa cases. It can also provide an insight into the functional and evolutionary consequences of epistasis.

We tested gene interactions between the two loci, rs4242382 at 8q24 and rs 10486567 at 7p, under the Fisher's model of statistical epistasis, and we found that the multiplicative model fitted the findings better than the additive model. This suggests the presence of linkage disequilibrium for these two loci [17]. However, a negative epistasis was found in the context of an additive model, whereas the multiplicative model suggested a positive epistasis. Different measurements for epistasis could lead to different interpretations; the model-dependence of joint effects has been well established in epidemiology and biostatistics [17]. In agreement with a previous study on the extension of common epistasis model with different classes of statistical models [17], we also found that adding different loci would yield different results. For example, in our additive model, an inclusion of rs10486567 did not change the effect of rs4242382, whereas adding rs4242382 substantially affected the influence of rs10486567. A similar phenomenon was observed in the multiplicative model. However, this epistasis was only observed in non-aggressive PrCa, but not in the aggressive PrCa, which suggests that the evaluation of the two loci cannot be used for identification of families with a risk of developing aggressive PrCa in the Finnish population. This may reflect the fact that both SNPs were originally found to be associated with PrCa risk only, not disease outcome, and that the later associations with disease outcome actually reflect other, yet unknown, and possibly population-specific interactions.

In conclusion, we proposed a family-based study design to demonstrate the effect of the previously reported SNP at 8q24, known as rs4242382, on the risk of multiple PrCa. Our findings suggest an interaction between rs4242382 and rs10486567 in both multiplicative and additive models. The proposed method is useful for identification of relevant variants in strong LD with the SNP of interest as well as quantifying epistasis between two loci affecting the penetrance of complex diseases and their traits.

## Supporting Information

File S1 Table S1, Likelihood ratio test for two loci with and without interaction models of prostate cancer. Table S2, Likelihood ratio test for two loci with and without interaction models of non-aggressive prostate cancer. Table S3, Likelihood ratio test for two loci with and without interaction models of aggressive prostate cancer.
(DOC)
