# Supplementary material for: Assessing Interactions of Two Loci (rs4242382 and rs10486567) in Familial Prostate Cancer: Statistical Evaluation of Epistasis
Source: PLoS One. 2014 Feb 25;9(2):e89508. doi: 10.1371/journal.pone.0089508 (PMC3934901; doi:10.1371/journal.pone.0089508)
Supplement: File S1 — Table S1, Likelihood ratio test for two loci with and without interaction models of prostate cancer. Table S2, Likelihood ratio test for two loci with and without interaction models of non-aggressive prostate cancer. Table S3, Likelihood ratio test for two loci with and without interaction models of aggressive prostate cancer. (DOC) [file pone.0089508.s001.doc]

Table S1. Likelihood ratio test for two loci with and without interaction models of prostate cancer

| Models for Testing(1) | AIC(1) | X^2^ | Models for Testing(2) | AIC(2) | d.f. | P-Value |
| --- | --- | --- | --- | --- | --- | --- |
| rs10486567 | 7516.3264 | 183.0093 | rs10486567|rs4242382 | 7699.3357 | 1 | <0.0001 |
| rs4242382 | 7682.4487 | 16.887 | rs4242382|rs10486567 | 7699.3357 | 1 | <0.0001 |
| rs4242382|rs10486567 | 7699.3357 | 13.8945 | rs4242382|rs10486567|Interaction | 7713.2302 | 1 | 0.0002 |

Table S2. Likelihood ratio test for two loci with and without interaction models of non-aggressive prostate cancer

| Models for Testing(1) | AIC(1) | X^2^ | Models for Testing(2) | AIC(2) | d.f. | P-Value |
| --- | --- | --- | --- | --- | --- | --- |
| rs10486567 | 4019.0588 | 49.3357 | rs10486567|rs4242382 | 4071.2496 | 1 | <0.0001 |
| rs4242382 | 4070.789 | 0.0865 | rs4242382|rs10486567 | 4071.2496 | 1 | 0.4973 |
| rs4242382|rs10486567 | 4071.2496 | 4.6477 | rs4242382|rs10486567|Interaction | 4075.1019 | 1 | 0.0497 |

Table S3. Likelihood ratio test for two Loci with and without interaction models of aggressive prostate cancer

| Models for Testing(1) | AIC(1) | X^2^ | Models for Testing(2) | AIC(2) | d.f. | P-Value |
| --- | --- | --- | --- | --- | --- | --- |
| rs10486567 | 453.152 |  | rs10486567|rs4242382 | 453.915 | 1 | 0.3824 |
| rs4242382 | 452.6681 | 0.0865 | rs4242382|rs10486567 | 453.915 | 1 | 0.2641 |
| rs4242382|rs10486567 | 453.915 | 4.6477 | rs4242382|rs10486567|Interaction | 454.2823 | 1 | 0.5445 |
